# Metabolism-driven glycosylation represents therapeutic opportunities in interstitial lung diseases

**DOI:** 10.3389/fimmu.2024.1328781

**Published:** 2024-03-14

**Authors:** Katarzyna Drzewicka, Zbigniew Zasłona

**Affiliations:** Molecure SA, Warsaw, Poland

**Keywords:** CHIT1, macrophage, IPF, sarcoidosis, metabolism, OATD-01, therapeutic targets

## Abstract

Metabolic changes are coupled with alteration in protein glycosylation. In this review, we will focus on macrophages that are pivotal in the pathogenesis of pulmonary fibrosis and sarcoidosis and thanks to their adaptable metabolism are an attractive therapeutic target. Examples presented in this review demonstrate that protein glycosylation regulates metabolism-driven immune responses in macrophages, with implications for fibrotic processes and granuloma formation. Targeting proteins that regulate glycosylation, such as fucosyltransferases, neuraminidase 1 and chitinase 1 could effectively block immunometabolic changes driving inflammation and fibrosis, providing novel avenues for therapeutic interventions.

## Pathogenesis of lung fibrosis and sarcoidosis – key cellular players

Interstitial lung diseases (ILDs) encompass over 200 conditions characterized by inflammation and fibrosis in the lung interstitium, which impairs gas exchange and can lead to respiratory failure in patients (1). This review focuses on two most common ILDs, pulmonary fibrosis and pulmonary sarcoidosis, both lacking curative treatments (2–4). The challenges in treating these diseases arise from their complex pathogenesis, involving a diverse range of cell types undergoing dynamic transitions and interactions (5, 6). This complexity primarily involves structural cells like epithelial cells and fibroblasts, as well innate immunity, including macrophages (7) and, to a lesser extent, neutrophils (8, 9) and adaptive immune cells (10, 11).

The current understanding assumes that in pulmonary fibrosis, damage to the epithelial layer initiates processes of both inflammation and tissue repair (10, 12). The causes of damage can vary and may encompass environmental factors (like cigarette smoke (13), irradiation (14)), genetic mutations (15) acute inflammation due to severe infections (like COVID-19) (16), or an unidentified cause, referred to as idiopathic pulmonary fibrosis (IPF). A critical point in the pathogenesis is when inflammation persists, particularly after severe injuries, leading to a dysregulated repair mechanism (10). This dysregulation is characterized by an excessive accumulation of extracellular matrix (ECM), culminating in fibrosis (10). The repair and fibrosis processes are primarily executed by fibroblast and epithelial cells, which not only proliferate to lock the wound but also undergo cellular transitions, namely epithelial-mesenchymal transition (EMT) (17) and fibroblast-myofibroblast transition (FMT) (18). These transitions result in the formation of ECM-producing myofibroblasts.

In the case of lung sarcoidosis, persistent infections or the presence of foreign materials can initiate the formation of structures called granulomas (6). Granulomas form due to the aggregation of macrophages, which can differentiate into epithelioid cells and then to multi-nucleated giant cells (6). A considerable number of cells within granulomas are CD4+ T cells (6). These cells are crucial to recognize antigen and trigger adaptive immune response. Sarcoidosis may progress to fibrosis at the granuloma sites, illustrating a complex interplay between ongoing inflammatory stimuli and fibrotic processes (19).

In ILDs, both inflammatory and resolution phase of inflammation and repair processes are primarily driven by macrophages (7, 20, 21). These cells can adopt various phenotypes and contribute to disease progression by secreting pro-inflammatory cytokines and pro-fibrotic factors. Moreover, crosstalk between macrophages and fibroblasts is needed for a successful process of a resolution of inflammation (22). Therefore, macrophages are recognized as central players in the pathogenesis of pulmonary sarcoidosis (6) and control of fibroblast transition and proliferation (23).

## Metabolic changes in pulmonary fibrosis and sarcoidosis

Most therapeutic approaches currently used in the clinic or being under development aim to target two major pathogenic processes in pulmonary fibrosis and sarcoidosis: inflammation or fibrosis (2, 3). Recent work focused on the pathogenesis of pulmonary fibrosis and sarcoidosis identified metabolic reprogramming, fostering glucose uptake and aerobic glycolysis (known as Warburg’s effect and previously associated with cancer), as a root cause of inflammation and fibrosis (24–27). Interestingly, this metabolic shift toward aerobic glycolysis occurs despite high oxygen concentrations present in the lungs (28). Although glycolysis generates significantly less energy compared to mitochondrial-driven oxidative phosphorylation (OXPHOS), it offers rapid energy production and serves as a source for various essential building blocks that fuel anabolic pathways (29). These pathways are activated in both lung diseases we focus on and include glycosylation, lipid, protein and nucleic acid synthesis (30, 31). Both inflammation (32, 33) and tissue repair (34–36) benefit from this metabolic switch, because they demand the extensive production of inflammatory mediators and extracellular matrix components, respectively. All key cellular transitions observed in these diseases, such as EMT (37), FMT (38) and granuloma-associated transitions (6, 39), require aerobic glycolysis. Glycolytic-driven activation of immune cells might contribute to epithelial damage in pulmonary diseases, for example via released cytokines such as tumor necrosis factor (TNF), causing loosening of tight junction and cell death (40, 41), which can be defined as a metabolic-driven injury.

## Increased glucose uptake as a marker of a pulmonary fibrosis and sarcoidosis

In fibrotic lungs, the transition to aerobic glycolysis becomes evident through increased expression of glucose transporter 1 (GLUT1) that was found on macrophages, facilitating the influx of glucose into cells (42). This transition is accompanied by elevated levels of glycolytic enzymes, including hexokinase (38, 43), phosphofructokinase 3 (PFKB3) (38, 44), pyruvate dehydrogenase kinase (45), and lactate dehydrogenase (43). Similarly, GLUT1 as well as total lung glycolysis measured by TLuG tool, increases during a process of granuloma formation in patients with pulmonary sarcoidosis (46). The significant rise in glucose uptake demanded by aerobic glycolysis, forms the basis for how positron emission tomography (PET) involving radiotracer 18-fluorodeoxyglucose (FDG), is effective in detecting and tracking sarcoidosis. In clinical trials, PET-CT was used as an endpoint read-out for monitoring sarcoidosis patients treated with antibodies against macrophage specific cytokines, such as Canakinumab (anti-IL-1β antibody) or CMK389 (anti-IL-18 antibody), from Novartis Pharmaceuticals (47). These studies demonstrate that FDG PET-CT scans for metabolic monitoring can be utilized to assess not only mentioned disease diagnosis and progression but also the efficacy of drugs that target macrophage activity and inflammation as their underlying mechanisms of action. Many novel approaches, being in the preclinical development, aim to target metabolic reprogramming to stop inflammation and fibrosis. For instance, inhibition of GLUT1 by a natural substance, phloretin, in macrophages, is found to be effective in mouse models of acute lung injury (48) and bleomycin-induced lung fibrosis (49). Moreover, anlotinib, a multitargeted tyrosine inhibitor drug, is found to reduce activation of PFKB3 and subsequently glycolysis during fibroblast activation (44).

## Glycosylation during homeostasis and disease

**Figure 1:**
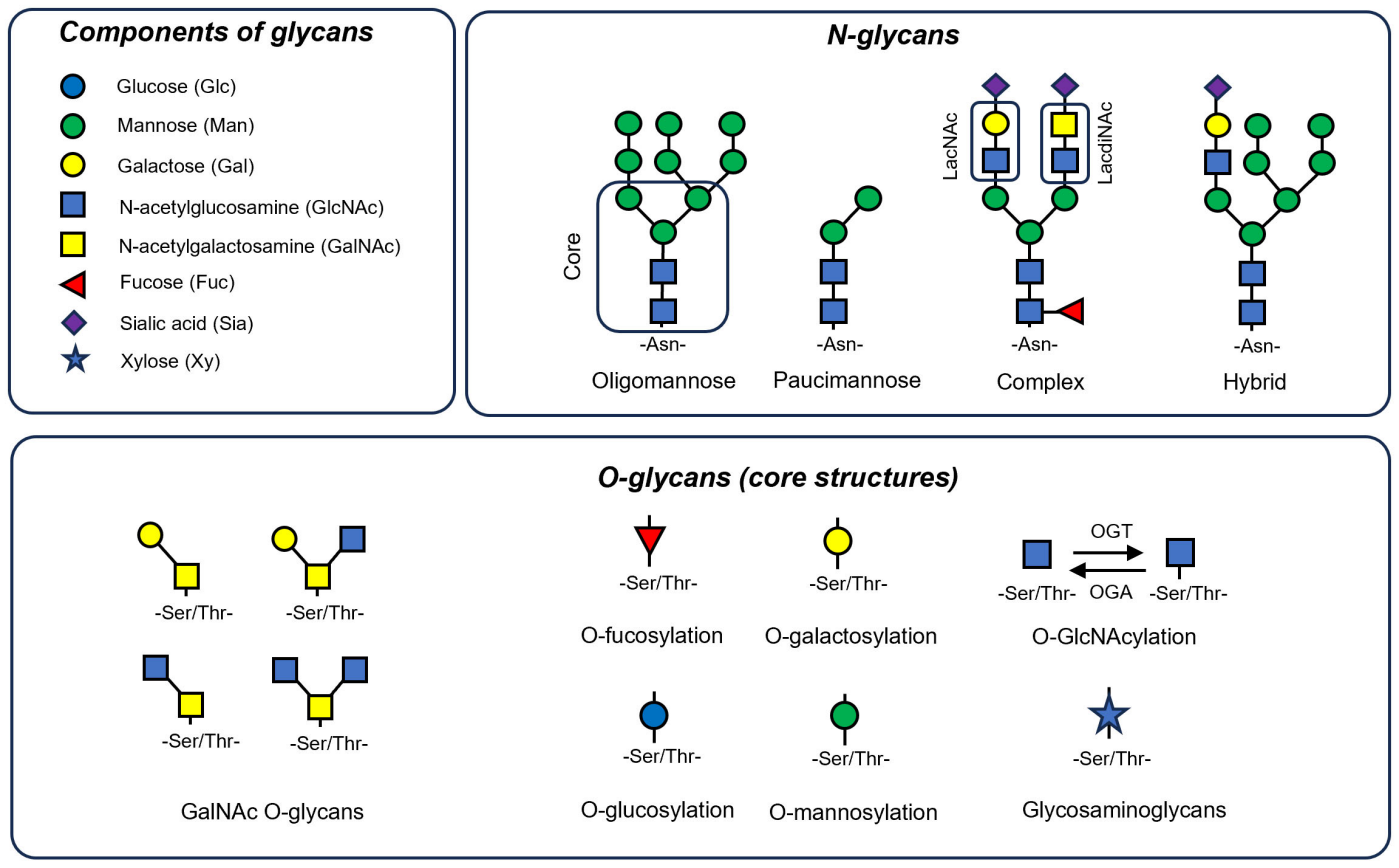
Components and basic structural forms of N-glycans and O-glycans. Most glycans are composed of monosaccharides like mannose (Man), galactose (Gal), N-acetylglucosamine (GlcNAc), N-acetylgalactosamine (GalNAc), fucose (Fuc), sialic acid (Sia) and xylose (Xy). N-linked glycans are attached to asparagine residues and are characterized by a core pentasaccharide structure, comprising two GlcNAc and three Man residues. These glycans are categorized into three principal types: oligomannose, which consists solely of Man residues in varying numbers branching off from the core; complex N-glycans, distinguished by their antennae that begin with a GlcNAc residue; and hybrid N-glycans, where both Man and GlcNAc residues extend as antennae from the core structure. Additionally, a type known as paucimannose can be identified, which bear only one or two Man residues. Here, two dissacharide units of N-glycans are markered. These are LacNAc (Galβ1-4GlcNAc) and LadiNACs (GalNAcβ1-4GlcNAc). O-linked glycans typically form through the attachment of GalNAc to Ser/Thr residues, resulting in various core types that can be further extended. Additionally, Ser/Thr residues can be linked to other monosaccharides like Fuc (O-fucosylation), Man (O-mannosylation), xylose (often the core for glucosaminoglycans), glucose (O-glucosylation) or GlcNAc (O-GlcNAcylation) that is the most dynamic modification performed by O-GlcNAc transferase (OGT) and O-GlcNAcase (OGA) enzymes.

**Figure 2:**
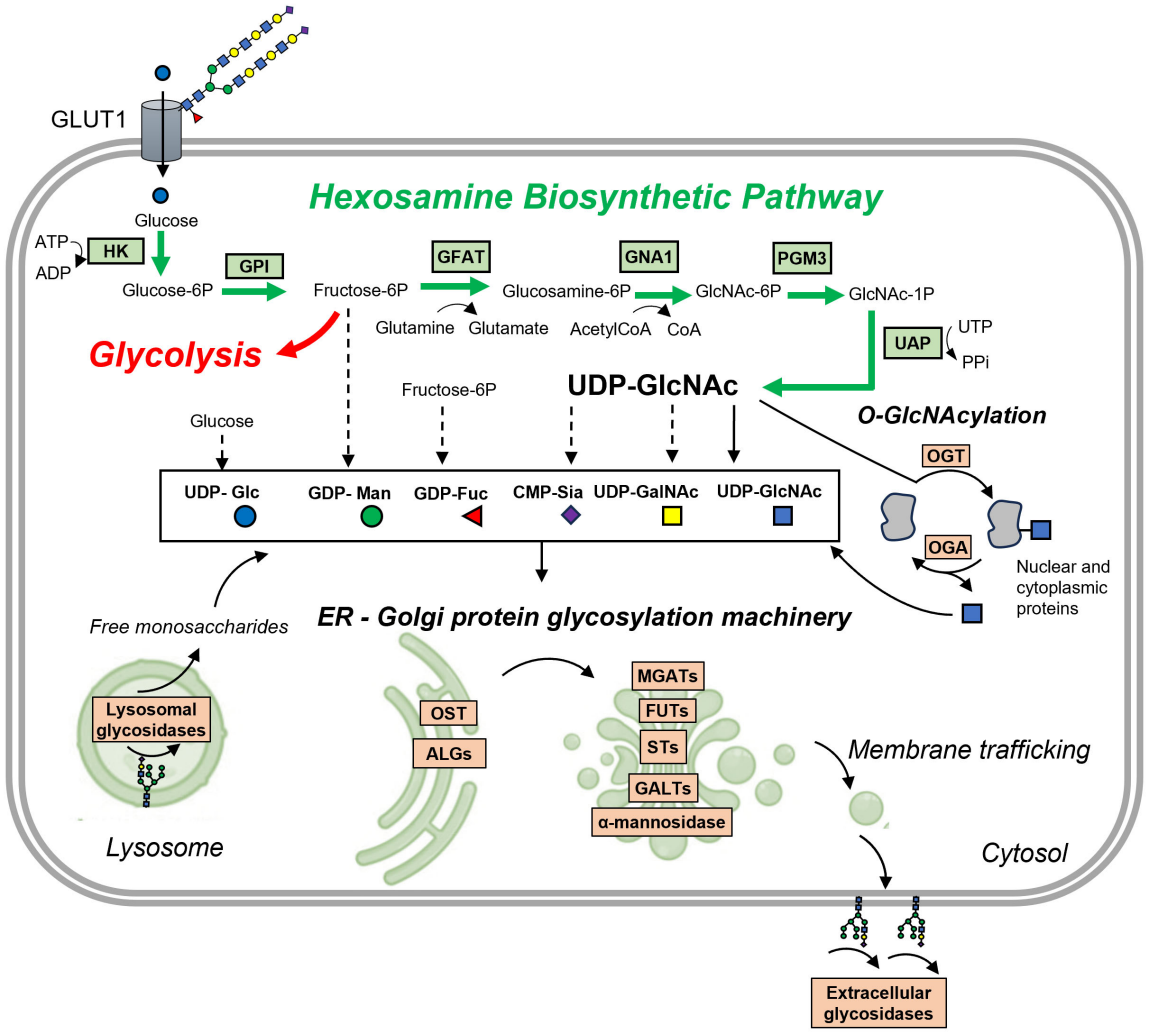
Protein glycosylation is closely interconnected with glycolysis and other metabolic pathways. Glucose is transported into the cell via the GLUT transporter, here GLUT1, which is known to be active in its glycosylated form (here with polyLacNAc glycans). The initial steps in converting glucose are shared between glycolysis and the hexosamine biosynthetic pathway (HBP). Here, glucose undergoes a two-step conversion: first, it is phosphorylated by hexokinase (HK) to form glucose-6-phosphate, and then, glucose-6-phosphate isomerase (GPI) transforms it into fructose-6-phosphate. The next critical step is catalyzed by glutamine:fructose-6-phosphate aminotransferase 1 (GFAT1), which converts fructose-6-phosphate and glutamine into glucosamine-6-phosphate and glutamate. This conversion is a rate-limiting step in the HBP. Following this, glucosamine-6-phosphate N-acetyltransferase (GNA1) converts glucosamine-6-phosphate into N-acetylglucosamine-6-phosphate, using acetylated-coenzyme-A from fatty acid oxidation (FAO). Phosphoglucomutase 3 (PGM3) then isomerizes this compound into N-acetylglucosamine-1-phosphate. Finally, UDP-N-acetylhexosamine pyrophosphorylase (UAP) synthesizes UDP-GlcNAc and diphosphate from N-acetylglucosamine-1-phosphate and uridine triphosphate (UTP). UDP-GlcNAc, along with other activated monosaccharides derived from UDP-GlcNAc, fructose-6-phosphate, or glucose, is utilized for protein glycosylation in the ER and Golgi by various glycosyltransferases (here ALGs, OST, MGATs, FUTs, STs, GALTs) and glycosidases (α-mannosidases). It is also involved in O-GlcNAcylation of nuclear and cytoplasmic proteins by O-GlcNAc transferase (OGT). O-GlcNAcase (OGA) catalyzes the removal of O-GlcNAc, adding back GlcNAc to the HBP pool for recycling through the salvage pathway. Besides OGA, some glycosidases located in the extracellular matrix contribute to remodeling glycosylated receptors or ECM proteins. Lysosomal glycosidases play an essential role in glycan turnover, as they hydrolyze existing glycans into monosaccharides that can be recycled for glycosylation. Other abbreviations: ALGs, various glycosyltransferases e.g. UDP-N-acetylglucosaminyltransferase (ALG13); MGATs, N-acetyglucosaminyltransferases; FUTs, fucosyltransferases; STs- sialyltransferases; GALTs, galactosyltransferases; OST, oligosaccharyltransferase.

Glycosylation, a process dynamically regulated by glycolysis, is changed during inflammation (50) and repair processes (51–54). Protein glycosylation is a post-translational modification, which can be broadly classified into two types: O-linked glycosylation, targeting serine or threonine amino acids, and N-linked glycosylation, which occurs on asparagine residues (55). At the molecular level, glycosylation generally increases protein stability (56), activity (57) and can change its localization (58, 59) and interacting partners (60, 61). Notably, a specific type of O-glycosylation called O-GlcNAcylation, primarily takes place intracellularly (in cytoplasm or nucleus) and regulates transcription factors and kinases signaling pathways, often competing with phosphorylation (62) (**Figures 1**, **2**). Protein glycosylation plays a pivotal role in a cell-cell and cell-ECM communication, being especially relevant for immune functions, where it modulates receptor-ligand interactions (63, 64) and receptor plasma membrane localization (59). In addition, changes in protein glycosylation are important to facilitate repair following injury, through processes like polarization of inflammation-resolving macrophages, EMT (51), epithelial migration and adhesion (52). For instance, N-glycosylation mediated translocation of annexin II to cell surface drives wound healing in airway epithelium (54). Similarly, alpha-dystroglycan that bind laminin in ECM was found to enhance airway epithelia wound healing (53). Alterations of these crucial functions of protein glycosylation are implicated in various disorders including ILDs, leading to the emergence of protein glycosylation as an important therapeutic target (65–67).

A good mechanistic example of a dysregulated glycosylation is a mutation in integrin α3 (ITA3) called A349S which has been identified in ILDs (68). This mutation leads to a gain-of-glycosylation and disrupts ITA3 biosynthesis, a crucial integrin highly expressed in lung epithelium playing a key role in IPF and the EMT (15). Another interesting study examined the alteration of N-glycans in irradiation-induced lung injury, a condition that frequently progresses to pulmonary fibrosis (69). This research utilized Matrix-Assisted Laser Desorption Ionization Mass Spectrometry Imaging (MALDI-MSI) to map the changes in N-glycans (69). The findings highlighted variations in different forms of N-glycans, which were localized to areas characterized by mucus presence, alveolar-bronchiolar hyperplasia, increased proliferation of epithelial cells, macrophage accumulation, edema, and fibrosis (69).

Dysregulated glycosylation has also been observed in the serum of pulmonary sarcoidosis patients, who have elevated levels of N-galactosylation of IgG4, which is a potential marker for this disease (70). Further examples of the importance of protein glycosylation as a functional biomarker and a driver of pulmonary fibrosis and sarcoidosis will be demonstrated in the context of glycan synthesis, regulation and its links to metabolic changes mainly in macrophages.

## Structure and synthesis of glycans

Glucose metabolism serves as a significant source of intermediates for glycan synthesis, followed by lipid and protein glycosylation. The synthesis of glycans is a highly complex process involving more than 200 enzymes, accounting for 10% of protein-encoding human genes (71). Glycans are built from monosaccharides such as mannose (Man), galactose (Gal), glucose (Glc), fucose (Fuc), xylose (Xy), N-acetylglucosamine (GlcNAc), N-acetylgalactosamine (GalNAc) and sialic acid (Sia), forming complex and variable combinations (an overview is presented in **Figure 1**) (55).

N-linked glycans typically have a pentasaccharide core structure that includes two N-acetylglucosamine (GlcNAc) and three mannose (Man) residues (72). These glycans can be classified into three main types: oligomannose, which have only Man residues of variable number branching off from the core; complex N-glycans, characterized by antennae starting with a GlcNAc residue; and hybrid N-glycans, where both Man and GlcNAc residues form extended antennae from the core (72). Additionally, so called paucimannose N-glycans can be distinguished, which are characterized by their unique structure of just one or two mannose residues, and represent an ancient type of glycan predominantly found in invertebrates (73). In vertebrates, these glycans are tissue and context specific, being enriched in pathological conditions like cancer (74) and inflammation (75) - their presence significantly increases in macrophages during infection (76). Additionally neutrophil elastase, a key secreted proteins in neutrophils, shaping ECM in lung fibrosis is rich in these glycans (77). O-linked glycans typically are formed through the attachment of GalNAc to Ser/Thr residues, resulting in various core types that can be further extended. Ser/Thr residues can be linked to other monosaccharides like Fuc (O-fucosylation) (78), Man (O-mannosylation) (79), xylose (initiation of glycosaminoglycans synthesis like heparan sulphate) (80, 81), Glc (O-glucosylation) (82) or GlcNAC (O-GlcNAcylation) (62).

Certain subunits of glycans are characterized by their affinity for specific lectins. For instance, disaccharides such as LacNAc (Gal-GlcNAc) (83) and the less common LacdiNAc (GalNAc-GlcNAc) (84) are found to interact with the galectin family of proteins (85). B4GALT1, an enzyme that synthesizes LacNAC, is found to be overexpressed in patients with IPF (86). Moreover, level of galectin 3 (GAL3), is increased in patients of pulmonary sarcoidosis (87) and fibrosis (88) and targeting GAL3 by small molecule inhibitors was showed efficacious in mouse models of bleomycin-induced lung fibrosis (89). Recent studies have linked LacNAc and LacdiNAc dissacharides with the process of granuloma formation, suggesting a pro-inflammatory role for these molecules (84) in IPF and sarcoidosis.

Glycosylation processes take place mostly in the endoplasmic reticulum (ER) and Golgi apparatus, where glycosyltransferases are responsible for glycan elongation, and glycosidases perform glycan trimming and remodeling (**Figure 2**) (55). The initial step in this process involves monosaccharides binding to a nucleotide sugar, creating an activated highly energetic form (e.g. UDP-GalNac, GDP-Fuc) that serves as a substrate for glycosyltransferases (55) (**Figure 2**). In the ER, the oligomannose type of N-glycans is initially attached to dolichol phosphate by various glycosyltransferases from ALG group and then transferred co-translationally to proteins by oligosaccharyltransferase (OST) (55). Subsequent modifications in the Golgi apparatus include trimming of Man by α-mannosidases and addition of various monosaccharides: Fuc by fucosyltransferases (FUTs), sialic acid by sialyltransferase (STs), GlcNAc by N-acetylglucosaminylltransferases (GnTs), Gal by galactosyltransferases (GalTs, including beforementioned B4GALT1), and GalNAc by N-acetylgalactosaminyltransferases (GalNTs) (55). In contrast, O-glycans are linked directly to synthetized protein in ER or most commonly in Golgi apparatus (55). Glycans can undergo additional modifications, such as acetylation and sulfation, adding further complexity and layers of regulation to their structure and function (90). Notably, after exiting ER-Golgi apparatus route, mature glycans can undergo fine-tuned remodeling by specific glycosidases (e.g. neuraminidase/sialidase, heparinase), which are found on the cell surface (91) and in the ECM (92, 93).

In addition to specific regulatory mechanisms, glycans can be broken down by a range of lysosomal glycosidases (94). These enzymes play a crucial role in hydrolyzing glycans, fragmenting them into monosaccharides (94). These monosaccharides can then be reused in glycan synthesis (94). The examples highlighted above demonstrate the strict regulation of glycosylation under normal physiological conditions. As a result, any disruptions in the glycosylation machinery can have significant adverse effects on health. Notably, ER stress is a recognized factor in the development of pulmonary fibrosis (95). The role of changes in protein glycosylation in relation to fibrotic alterations in the context of ER stress has been investigated by Lee and colleagues (96). Their research demonstrates that BAX inhibitor-1, which acts as a negative regulator of ER stress, enhances the glycosylation of lysosomal V-ATPase (96). This modification leads to increased activity of lysosomal glycosidases that break down collagen glycan, thereby contributing to the degradation of collagen. This process is crucial in reducing collagen deposition, as shown in cellular assays and in an *in vivo* model of bleomycin-induced lung fibrosis (96).

## Protein glycosylation is linked to metabolism

The main building block for glycans is uridine diphosphate N-acetylglucosamine (UDP-GlcNAc), synthesized in the hexosamine biosynthetic pathway (HBP) (97) (**Figure 2**). This pathway requires several substrates such as fructose-6-phosphate (from glucose metabolism), glutamine (from amino acids metabolism), acetyl-CoA (from fatty acids metabolism) and UDP (from nucleotide metabolism). Hence, UDP-GlcNAc is known as a sensor of the metabolic state in cells using components of all four macromolecules. Concentration of these substrates, as well as levels and activities of glycosyltransferase enzymes will affect dynamics of HBP pathway and subsequent glycosylation. For instance, influx of UDP-GlcNAc affects glycan branching that is mediated by β-1,6-N-acetylglucosaminyltransferases (MGATs: MGAT1, 2, 4 and 5) (98). These enzymes have varying affinities towards UDP-GlcNAc. In situations where UDP-GlcNAc is limited, only MGAT1, which has the highest affinity, is active, leading to reduced glycan branching (98). Conversely, when UDP-GlcNAc is abundant, it activates other MGAT enzymes, resulting in glycans with more extensive branching (98).

UDP-GlcNAc can be then further converted into other building blocks of complex glycans, including GalNAc and sialic acid (99). Mannose, fucose and galactose can be derived from fructose-6-phosphate, directly depending on glucose influx (99). HBP plays a crucial role in glycan biosynthesis, converting glucose into UDP-GlcNAc through a six-step process that partially overlaps with glycolysis. This process begins with the phosphorylation of glucose by hexokinase (HK), producing glucose-6-phosphate. Subsequently, glucose-6-phosphate isomerase (GPI) transforms glucose-6-phosphate into fructose-6-phosphate. The next critical step, catalyzed by glutamine:fructose-6-phosphate aminotransferase 1 (GFAT1), involves the conversion of fructose-6-phosphate and glutamine into glucosamine-6-phosphate and glutamate, a rate-limiting step in the HBP. Following this, glucosamine-6-phosphate N-acetyltransferase (GNA1) converts glucosamine-6-phosphate into N-acetylglucosamine-6-phosphate, utilizing acetylated-coenzyme-A from fatty acid metabolism (FAO). Phosphoglucomutase 3 (PGM3) then isomerizes N-acetylglucosamine-6-phosphate into N-acetylglucosamine-1-phosphate. Finally, UDP-N-acetylhexosamine pyrophorylase (UAP) synthesizes UDP-GlcNAc and diphosphate from N-acetylglucosamine-1-phosphate and uridine triphosphate (UTP). Crucial enzymes within the HBP pathway such as (GFAT1, GNA1, PGM3) are elevated in a process shared by various ILDs, namely when epithelial-mesenchymal plasticity (EMP) is induced by TGF-β (100). This elevation is necessary for the N-glycosylation process and subsequent secretion of extracellular matrix (ECM) components, which is vital for airway remodeling, highlighting fibrosis-promoting role of the HBP pathway.

## Macrophages have wide spectrum of phenotypes and metabolism

Having in mind recent advances in a field of immunometabolism, we would like to explore in detail the glycosylation process and its link to metabolism in a major cell type of innate immunity. Macrophages play a pivotal role in the pathogenesis of pulmonary fibrosis and sarcoidosis due to their plastic metabolism (101, 102). For simplicity, these cells are often broadly classified into M1-like and M2-like phenotypes, each having distinct roles (103). Classically, M1-like macrophages active during inflammation heavily rely on aerobic glycolysis for producing cytokines and chemokines (103). In contrast, M2-like macrophages shift towards OXPHOS-driven metabolism during resolution of inflammation and tissue repair process (103). Highly plastic metabolism allows macrophages to switch between different phenotypes (104). It has been shown that alteration of metabolic pathways associated with aerobic glycolysis can shift from M1-like towards M2-like (104). This reprogramming is unique to macrophages, since other immune cells from innate and adaptive immune system have limited abilities to change their phenotype once they have specialized (105, 106). Furthermore, macrophages, with a lifespan ranging from months to years, possess the opportunity to shape microenvironment both within an afflicted lung and during the recovery phase (107, 108). The immunometabolic profile of macrophages is influenced not only by the microenvironment but also their origins (109). For example, contrary to expectations, tissue-resident alveolar macrophages transition towards a pro-inflammatory state following injury, heavily depending on OXPHOS respiration, as treatment with glycolysis inhibitor, 2-deoxyglucose (2-DG), has almost no effect on secretion of cytokines (109). This underscores the broader array of potential immunometabolic states within the realm of macrophages.

## Glycosylation regulates macrophage phenotype

In activation states macrophages undergo metabolic changes that precede their pathogenic shift into either an inflammatory or fibrotic phenotype. These metabolic alterations are primarily directed by the mammalian target of rapamycin - hypoxia-inducible factor 1α (mTOR-Hif1α) axis (103, 110, 111). Specifically, in the M1-like pro-inflammatory state, the mTOR-Hif1α axis is activated (110). This results in an increase in GLUT1 expression, augmented glucose uptake, glycolysis (112) and activation of anabolic pathways like pentose phosphate pathway to sustain infammation (113). Conversely, in the M2 state, there is a suppression of the aforementioned pathways, and mitochondrial metabolism based on OXPHOS and fatty acid oxidation (FAO) becomes dominant (103). Importantly, these metabolic shifts also affect glycosylation processes, which serves a dual role - it can be both an executor and a regulator of immunometabolic alterations within macrophages. The link between metabolism and glycosylation is especially evident in protein O-GlcNAcylation, an intracellular process where UDP-GlcNAc, a product of HBP, is added to serine or threonine residues (62). This modification is very dynamic being facilitated by the OGT enzyme and reversed by the OGA enzyme (62) (**Figures 1**, **2**).

The role of O-GlcNAcylation in macrophage polarization is complex and sometimes contradictory. Various studies using inhibition of O-GlcNAcylation by GlcNAc or genetic deletion of OGA or OGT enzymes, suggest that O-GlcNAcylation favors the M1-like phenotype by modifying and activating crucial pro-inflammatory transcription factors such as p65 (114), c-Rel (115), and STAT3 (116) (see **Table 1**). Conversely, it has been shown that HBP activity and protein O-GlcNAcylation decreases in LPS-treated macrophages (61). In the same study, inhibiting protein O-GlcNAcylation by deletion of OGT enzyme enhances immune response and necroptosis, by reducing RIPK3 O-GlcNAcylation and inhibiting its interaction with RIPK1 (61). In line with this, deletion of OGT enzyme shifts macrophages towards M1-like phenotype (138). Interestingly, increased O-GlcNAcylation is observed in M2-like macrophages (139). This can be attributed to the increased availability of HBP substrates including acetyl-CoA from the TCA cycle and glutamine, which is boosted by enhanced glutaminolysis and increased glutamine uptake in M2-like macrophages (140). We think that the contradictory data on the role of O-GlcNAcylation in macrophage polarization may be result of dynamic nature of this modification that relays on metabolite influx and may vary in different models. Moreover, depletion of an enzyme in the O-GlcNAcylation cycle may not necessarily lead to a global loss or gain of O-GlcNAcylation; instead, it could selectively affect specific targets, as observed in other types of reversible protein modifications (141). Several proteins governing macrophage metabolism-driven regulation, including mTOR (142) and the AMP-activated protein kinase (AMPK) (143), have been found to undergo O-GlcNAcylation. Investigating macrophage O-GlcNAcylation status could provide valuable insights into macrophage function and polarization.

Table 1Glycosylated proteins in macrophages implicated in progression if inflammation and fibrosis as well as glycosidase targets and their inhibitors at different stages of development for treating pulmonary fibrosis and sarcoidosis.

**Table:** 

Glycosylation of macrophage proteins that promotes inflammation			
Protein	Type of glycosylation		References
p65	O-GlcNAcylation		(114)
c-Rel	O-GlcNAcylation		(115)
STAT3	O-GlcNAcylation		(116)
TNFR1	N-glycosylation		(117)
CD14	N-glycosylation: oligomannose and complex types		(59, 118, 119)
CD147	N-glycosylation: oligomannose and complex types		(120, 121)
TLR2	N-glycosylation		(59, 122)
TLR7	N-glycosylation		(59)
Glut1	N-glycosylation: oligomannose and complex types with polyLacNAc		(58, 123–126)

**Table:** 

Glycosylation of macrophage proteins that promotes fibrosis			
CD206	N-glycosylation:complex type and O-glycosylation		(60)
CD301	N-glycosylation		(127)
integrin β1	N-glycosylation		(128, 129)
integrin β2	N-glycosylation		(130)

**Table:** 

Glycosidase targets and their inhibitors for treating pulmonary fibrosis and sarcoidosis			
Target	Small molecule	Stage of development	References
NEU1	C9-BA-DANA	Pre-clinical	(131)
FUTs	fucose mimetics	Pre-clinical	(132)
CHIT1	OATD-01	phase II for pulmonary sarcoidosis, study number: NCT06205121	(133–137)

Conversely to O-GlcNacylation, N-glycosylation is found mostly in secretory proteins as well as on membrane receptors, shaping interactions between macrophages and ECM as well as fibroblasts (144). Inhibition of N-glycosylation by tunicamycin (UDP-GlcNAc analog) lowers down the expression of many M2-like activation markers including two cell surface lectins CD206, and CD301 (127) both relevant to progression of fibrosis (145) and sarcoidosis (146–148) (see **Table 1**). Both lectins are engaged in recognition, endocytosis and presentation of glycosylated proteins (149, 150) as well as adhesion and fusion during granuloma formation (147, 148). In case of CD206, commonly known as mannose receptor, it is shown that glycosylation status of this receptor is influencing its binding to mannose, showing that glycosylation can also alter lectin-glycan interactions (60). In M1-like macrophages, tunicamycin causes loss of TNF receptor 1 (TNFR1) N-glycosylation and inhibits pro-inflammatory response (117) (see **Table 1**). In another study, authors using N-glycoproteomic profiling of murine macrophages stimulated with LPS and viruses such as HSV and VSV, showed that N-glycosylated proteins were mostly linked to immune functions such as antigen processing and presentation, as well as cytokine secretion (59). Among N-glycosylated proteins were TLR2, TLR7 and CD14, receptors crucial for pathogen recognition and macrophage activation (59) (see **Table 1**).

Noteworthy, GLUT1 a receptor elevated in M1-like state, is also N-glycosylated and this modification promotes its plasma membrane localization, stability, and glucose uptake (58, 123–125, 151). Studies on other cell types, including erythrocytes and cancer cells, show that GLUT1 possess oligomannose and complex N-glycans rich in poly-LacNAc (125, 126) (see **Table 1**). Potentially, activity of extracellular glycosidase that can process some of these saccharides could affect GLUT1 activity and in consequence metabolism. N-glycosylation of GLUT1 is a perfect example of glycosylation directly regulating metabolism, and specifically glucose uptake during glycolysis, which eventually determines immune response.

Another N-glycoprotein important for macrophage phenotype is CD147/EMMPRIN, a plasma membrane receptor. Glycosylation level of CD147 determines its functionality, which is primarily to activate expression of various matrix metalloproteases (MMPs), including MMP-1, MMP-2, MMP-3, MMP-9, and MMP-14 (120, 121, 152) (see **Table 1**). Apart from inducing MMPs, macrophage -expressed CD147 can stimulate its proinflammatory phenotype (153). In THP1 cells, highly glycosylated form of CD147 increases after stimulation with pro-inflammatory activators and induces adhesion, migration, ERK, and NF-κβ signaling (153). Recent data strongly implicates CD147 as a driver of pulmonary fibrosis (152) including the one induced by SARS-CoV2 (154). CD147 blockage by antibody diminishes M1-like phenotype and in consequence reduced Th17 cell differentiation in bleomycin-induced lung fibrosis (155). Altogether, O-GlcNAcylation and N-glycosylation of proteins are crucial to drive or regulate immunometabolic changes in macrophages.

## Differences in glycan structures between different macrophages

Macrophages can accumulate in tissues by local proliferation or recruitment from circulating monocytes followed by a differentiation process (156). Glycomic and glycoproteomic techniques have been used to identify glycans and glycosylated proteins during the differentiation of monocytes into macrophages (157) and their subsequent polarization (59, 158). Study from Hinneburg et al., 2020 found that both blood-derived human monocytes and macrophages possess typical to innate immunity ancient mannose-terminating N-glycans (paucimannosidic/oligomannosidic type) as well as trimming machinery of intracellular glycosidases possibly involving N-acetyl-β-hexosaminidases and α-mannosidases that produce such short structures (157). Although the presence of these specific glycans in macrophages has been confirmed in another study involving THP1 cells (76), their role in macrophage function, specifically in the context of pulmonary fibrosis and sarcoidosis, is not yet understood. The levels of paucimannosidic and oligomannosidic glycans as well as core-fucosylation is found to be slightly reduced in macrophages compared to monocytes (157). More pronounced differences in glycosylation are evident on the macrophage cell membrane, marked by elevated levels of mannosidic and sialylated glycans, which intensify as maturation progresses (157). Observation from Park et al., 2021 highlights a considerable difference in the glycan profile between resident and newly recruited to the tissue macrophages (158). This suggests that glycosylation is not solely dependent on the macrophage’s phenotype but is also influenced by its origin.

While examining the differences between M1-like and M2-like macrophages, some significant changes in specific structures of O- and N-glycans were identified (158), which in the future may help to further characterize macrophage phenotypes. Alterations of protein glycosylation in macrophages, specifically expression changes of glycosyltransferases and sulfotransferases during the monocyte-macrophage transition and in response to LPS stimulation, have been reported (159). These findings indicate that the expression of genes encoding glycosyltransferases (e. g. ALG9, FUT8) and sulfotransferases (e. g. ST6GALNAC3) increases during differentiation of macrophages while being down-regulated during M1 activation, suggesting significant changes in protein glycosylation in major processes of macrophage biology (159). Furthermore, Delannoy and colleagues have found that the transcript levels of key glycosylation enzymes significantly increase during the differentiation of THP-1 macrophages, including ST3GAL5, MGAT1, MGAT5, B4GALT1, FUT8, and NEU1 (160). In fact, macrophage maturation might represent the period of most significant increase in glycosylation and further studies need to confirm this.

In conclusion, while the distinct metabolic states of monocytes, macrophages and their M1 and M2 subtypes are characterized by a specific glycosylation patterns, it is vital to emphasize the importance of the macrophage’s origin in influencing these outcomes. Further studies concerning glycan structure on macrophages will help to identify specific subsets of innate immunity cells and possibly track their origins, since glycosylation patterns are evolutionary conserved. With better molecular biology tools determining glycan profile of immune cells, we will be able to predict diseases progression and more accurately distinguish between the inflammatory and resolution phases of various disorders.

## Glycosylation of integrins regulates macrophage biology in ILDs

In the context of pulmonary fibrosis and sarcoidosis, a critical aspects of macrophage biology worth discussing include adhesion (161, 162), phagocytosis (163, 164), and tissue migration (165). All of these processes are mediated by the integrins – a protein family whose function depend on glycosylation status (166, 167). Integrins are glycoproteins, consisting of α and β subunits, which are considered as therapeutic targets for cancer and respiratory diseases, including pulmonary fibrosis and sarcoidosis (168–170). Both subunits require N-glycosylation for the formation of heterodimers, proper plasma membrane localization, and effective interactions with extracellular matrix proteins (e.g. fibronectin, collagen) and cellular ligands (128, 129, 171, 172). The extent of N-glycosylation varies depending on the specific integrin receptors, and these variations are linked to distinct adhesive properties (128, 129). This is evident for two common macrophage integrin complexes, α4β1 and α5β1 that bind to fibronectin (128, 129, 173). It was found that integrin α4β1 binding to fibronectin can be hindered by accelerating the N-glycosylation processing of the integrin receptor (129), while an increase in the N-glycosylation state of α5β1 enhances cell adhesion (128).

Integrins are known to be modified by LacdiNAcs disaccharides, that are found at the tips of complex N- or O-glycans (174) (**Figure 1**). These disaccharides have the ability to modify the adhesive properties of integrins and research has predominantly concentrated on their impact in the context of cancer cells (174, 175). Interestingly, integrin complexes sense the “stiffness” of ECM and in turn switch on signaling cascade that influence mitochondrial metabolism (176). Mitochondria can also impact integrins’ ability to bind to their ligands, highlighting the phenomenon of a feedback loop between protein glycosylation and metabolic alternations (177). Specifically, dysfunction in OXPHOS can lead to increased glycosylation of β1 integrin, thereby promoting its binding to its ligand (177).

Integrins are known to contribute to the onset and progression of pulmonary fibrosis and sarcoidosis (169, 178). Mechanistic insights include work where alveolar M2-like macrophage attachment to collagen type I was facilitated by β2-integrins, which are known to be heavily glycosylated (130), leading to an increased CCL18 production – a biomarker of pulmonary sarcoidosis (179). This interaction creates an ongoing cycle of enhanced M2-like macrophage activation and excessive collagen production by lung fibroblasts (179). In pulmonary sarcoidosis increased expression of β1- and β2-integrin complexes on sarcoid monocytes and macrophages is a hallmark of the disease, by contributing to enhanced phagocytosis and antigen presentation by these cells (180, 181). Currently, integrins are targeted by antibodies blocking their functions and developed as therapeutics for IPF (182). It would be interesting to study if glycosylation of integrins is linked with metabolic changes altering macrophage functions that contribute to progression of ILDs. Therefore, future studies addressing glycosylation state of integrins can present novel therapeutic opportunities.

## Proteins regulating glycans are promising therapeutic targets in ILDs

So far, we have presented examples of crucial proteins involved in the development of ILDs whose functions can be altered by targeting their glycosylation status. Another approach is to focus on proteins capable of sugar modifications like adding specific sugar residue or glycan hydrolysis. Specifically, we want to highlight glycan-regulating proteins linked to macrophages, which hold promise as therapeutic targets in pulmonary fibrosis and sarcoidosis using as examples fucosyltransferases (FUTs), neuraminidase-1 (NEU1), and chitinase-1 (CHIT1) (see **Table 1**).

## FUTs

Fucosylation mediated by FUTs can alter receptor-ligand interactions (183); typically, fucosylated epitopes are bound by selectins, which are important for leukocyte adhesion (183). FUTs can add a fucose residue either at terminus or at the core of a glycan (183). Both types of modification are involved in driving inflammatory phenotype of macrophages. Inhibition of terminal FUTs, like FUT1/3/7/9 that are upregulated upon inflammatory stimulus, leads to a shift in M1-like differentiation toward M2-like macrophages (184). In addition, deletion of FUT8 involved in core fucosylation diminished inflammation in macrophages by CD14 regulation of TLR2 and TLR4 (118). It was also found that expression of FUT8 is increased after monocyte to macrophage differentiation (159). FUT8 is already considered as a therapeutic target in IPF, because of its role in enhancing crucial signaling for IPF such as TGFβ (185) and IGF (186, 187). Better understanding of the role of fucosylation and FUTs in pulmonary fibrosis and sarcoidosis should unravel new biomarkers and present therapeutic targets. Fucosylation has been targeted with fucose mimetics, but so far only in the context of cancer indications (132). Ideally, more specific synthetic inhibitors could provide better efficacy in ILDs.

## NEU1

NEU1 belongs to the NEU family of neuraminidases/sialidases that cleave off the terminal sialic acid residue from glycan moiety (188). This, in turn, can change receptor-ligand interactions since sialic acid is recognized typically by cell surface proteins called Siglecs which regulate adhesion, antigen recognition and presentation (189). Increased expression of NEU1 is observed in the lungs of patients with IPF, and NEU1 participates in the pathogenesis of lung fibrosis by provoking lymphocytic infiltration and promoting accumulation of glycoprotein TGFβ, type I and III collagen (190). In macrophage biology, NEU1 localizes at the plasma membrane during monocyte to macrophage differentiation (191), where it is crucial for phagocytosis and antigen presentation (191, 192) - processes especially relevant in granuloma formation. NEU1 has been demonstrated to induce a pro-inflammatory phenotype in macrophages by activating TLR receptors through desialylation (193). Most recent study presents NEU1-selective inhibitor, C9-BA-DANA, mimetic of sialic acid, that dose-dependently inhibited bleomycin-induced lung fibrosis in mouse models (131), presenting its therapeutic potential for IPF patients.

## CHIT1

CHIT1 is a macrophages specific enzyme, belonging to the GH18 glycosidase family, cleaving β(1→4) glycosidic bonds in glycans, which play a significant role in various lung diseases (194). CHIT1 is capable of processing LacNAc and LacdiNAc (195), suggesting its direct role in modification of protein glycosylation. This enzyme is primarily known as an extracellular glycosidase, but literature suggests its presence in the form of catalytic domain in the lysosomes of macrophages, pointing to a potential intracellular role for the enzyme (196). Our data present CHIT1 as a promising therapeutic target capable of modification of the glycosylation of proteins, which leads to an overall anti-inflammatory and anti-fibrotic effects in various pre-clinical models. Intriguingly, insights from a single-cell RNAseq lung atlas revealed that CHIT1 is exclusively expressed within a specific subset of emerging fibrosis-specific macrophages in the lungs of patients with IPF (133). In the context of pulmonary sarcoidosis in another un-biased study, CHIT1 expression was observed in activated macrophages within granulomas - confirmed through transcriptomic data and immunocytochemistry (134). CHIT1 expression increases during monocyte-macrophage differentiation (197). Macrophages treated with 2-deoxyglucose exhibit reduced CHIT1 expression (198), revealing a link between the process of glycolysis and CHIT1 induction. CHIT1 is a well-known biomarker in lung sarcoidosis, which correlated with diseases severity and progression (199). The CHIT1 inhibitor, synthetic drug OATD-01, targeting GH18 glycosidase active site, effectively mitigates inflammation driven by macrophages and indirectly influences fibroblast behavior, leading to decreased collagen deposition and a lowered fibrotic score in *in vivo* model of bleomycin-induced lung fibrosis (133). Similarly, OATD-01 suppresses granuloma formation in a murine MWCNT + ESAT6 sarcoidosis model, while modulating immune responses in macrophages (134). OATD-01 has demonstrated efficacy in multiple models characterized by chronic inflammation and fibrosis including pulmonary fibrosis (133), non-alcoholic steatohepatitis (135), inflammatory bowel disease (200) and chronic asthma (201) – diseases where pathological macrophages contribute to chronic inflammation leading to fibrotic changes. First-in-human proof-of-concept phase II clinical study assessing efficacy of OATD-01 in patients with pulmonary sarcoidosis will use F-FDG PET/CT imaging as a primary endpoint (study number: NCT06205121). This readout utilizing an analogue of glucose, will provide valuable functional information of OATD-01 based on the increased glucose uptake and glycolysis of granulomas, reflecting inflammatory activity of granulomas in the lungs of sarcoidosis patients.

## Summary and future perspectives

**Figure 3:**
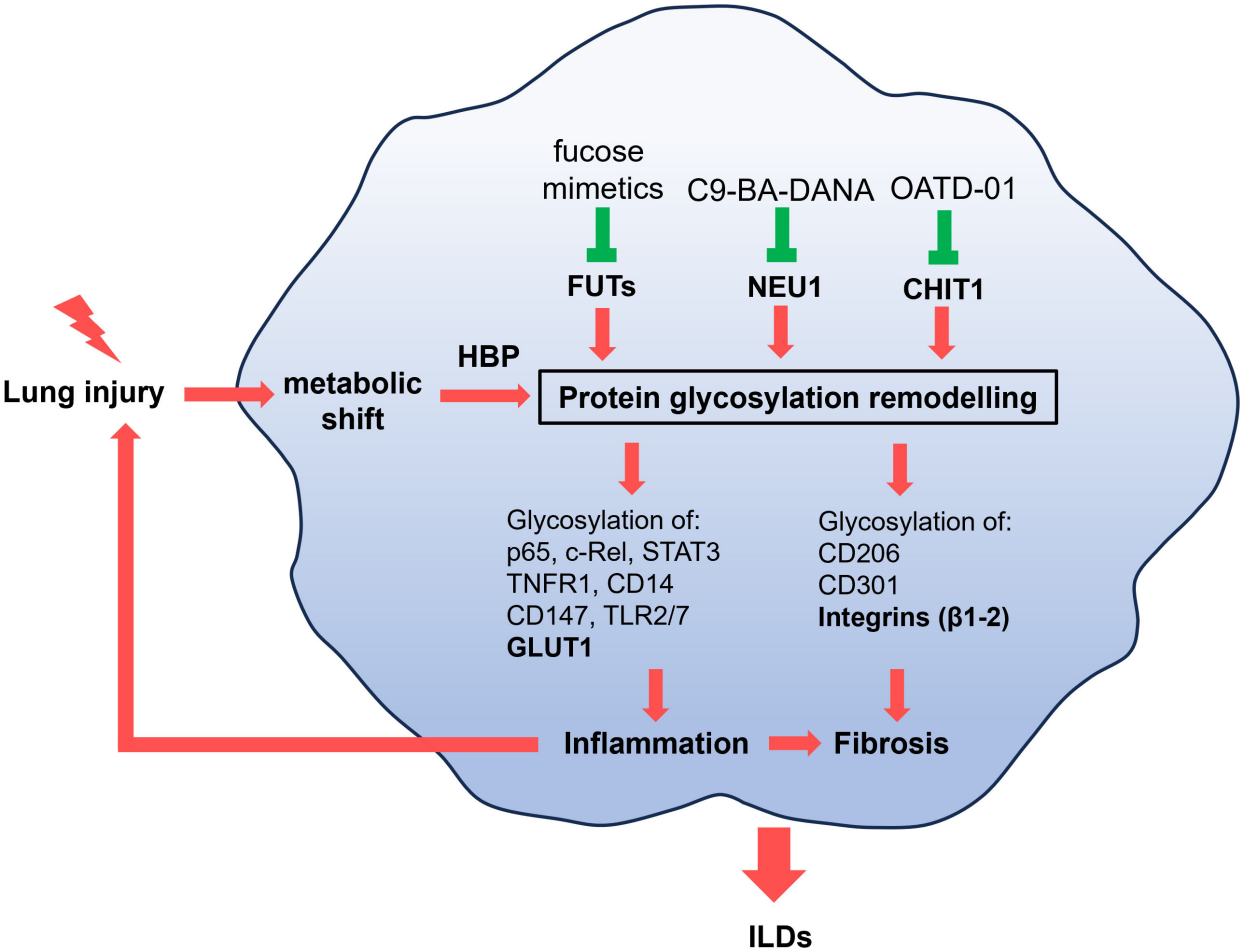
Pathological macrophages that promote inflammation or fibrosis have altered metabolism as a results of lung injury. The metabolic shift, through hexosamine biosynthetic pathway (HBP), causes changes to the glycosylation of proteins implicated in inflammation (p65, c-Rel, STAT3, TNFR1, TLR2, TLR7, CD14, CD147, GLUT1) or fibrosis (CD206, CD301, integrins β1-β2). Inflammation can perpetuate injury to the lung and promote fibrotic changes. These alterations drive the progression of interstitial lung diseases (ILDs), including pulmonary fibrosis and sarcoidosis. While metabolism can be regulated by key glycoproteins such as GLUT1 and integrins (bolded), known therapeutic targets in ILDs, we propose a novel approach: targeting the glycans regulating proteins such as CHIT1, NEU1, and FUTs to address metabolic-driven glycosylation in ILDs.

Inflammation and fibrosis are lung diseases with unmet clinical need that have long been in the center of the search of new therapeutics. Recently, the advances in the area of pharmaceutical exploitation of a metabolic switch that drives both inflammation and fibrosis opened new opportunities. In this review, we highlight the process of protein glycosylation as a therapeutic strategy, and provide evidence of how it regulates immune-metabolic changes (**Figure 3**). We used macrophages as cells where biological consequences of altered protein glycosylation was successfully studied and focused on ILDs – incurable diseases where patients would significantly benefit from new research.

Although the literature provides numerous examples of alterations in protein glycosylation or changes in the expression of proteins that regulate glycosylation in pulmonary fibrosis and sarcoidosis, there remains an obvious need for a comprehensive glycan profiling and glycoproteomic studies. These studies are essential for identifying novel biomarkers and key factors altering function of crucial proteins driving the progression of ILDs. Studies that combine glycoproteomics with metabolomics, specifically focusing on macrophages, are particularly valuable. This integrated approach would enable a further exploration leading to therapeutic use of the glycosylation and metabolism. Currently, these types of analyses are technically challenging, and there are limited methods to directly compare levels of different glycan structures across various disease conditions. Therefore, we believe that the use of glycobiology field in drug discovery is limited by technical restrictions rather than biological potential. Specifically since glycosylation is highly sensitive to metabolic fluctuations that can lead to data inconsistencies.

Glycosylation patterns depends highly on metabolism and glycosylation influences metabolic changes. Key examples of glycosylated proteins explored in this review include GLUT1 and integrins. We discussed the role of HBP, which serves as a link between metabolism and glycosylation justifying further exploration in lung fibrosis and sarcoidosis. Finally we present FUTs, NEU1 and CHIT1 as enzymes regulating glycosylation in various inflammatory and fibrotic diseases. As often in drug discovery new translational studies can only advance the field when a basic research presents clear scientific perspective. The focus on exploration of novel glycan-regulating proteins influencing metabolism has a potential to identify new drugs for ILDs.

## Author contributions

KD: Writing – original draft. ZZ: Writing – original draft.
